# Supplementary material for: CRISPRi-based functional genomic screening identifies genes essential for CH4-dependent growth in a methanotrophic bacterium
Source: Sci Adv. 2026 Apr 24;12(17):eaed4234. doi: 10.1126/sciadv.aed4234 (PMC13108547; doi:10.1126/sciadv.aed4234)
Supplement: Supplementary file 1 — Figs. S1 to S3 Legends for tables S1 to S17 Tables S18 and S19 [file sciadv.aed4234_sm.pdf]

Science Advances
AAAS

Supplementary Materials for

CRISPRi-based functional genomic screening identifies genes essential for CH_4-dependent growth in a methanotrophic bacterium

Jessica M. Henard *et al.*

Corresponding author: Calvin A. Henard, Calvin.Henard@unt.edu

Sci. Adv. **12**, eaed4234 (2026)
DOI: 10.1126/sciadv.aed4234

The PDF file includes:

Figs. S1 to S3
Legends for tables S1 to S17
Tables S18 and S19

Other Supplementary Material for this manuscript includes the following:

Tables S1 to S17

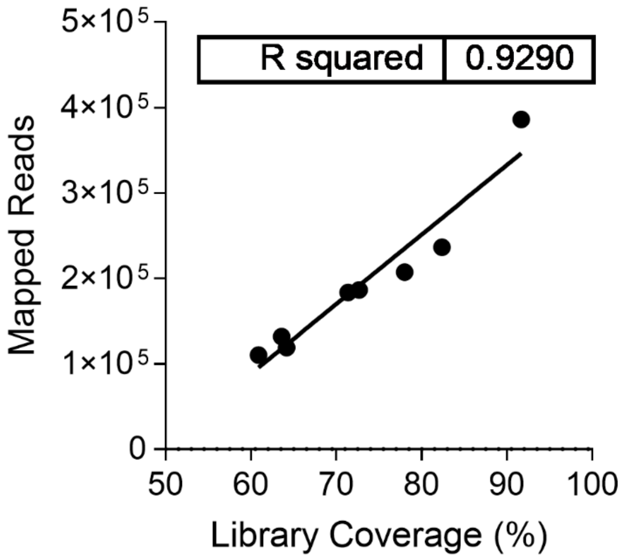

Figure S1. The relationship between sequencing depth and sgRNA library coverage. Oxford Nanopore Technology sequencing reads were processed to extract sgRNA target sequences that were mapped to the synthesized genome-wide sgRNA library. Mapped sequence reads varied between samples. Linear regression analysis suggests 413,960 mapped reads are needed to achieve 100% sgRNA library inclusion in downstream analyses, which is ~10x sequencing depth.

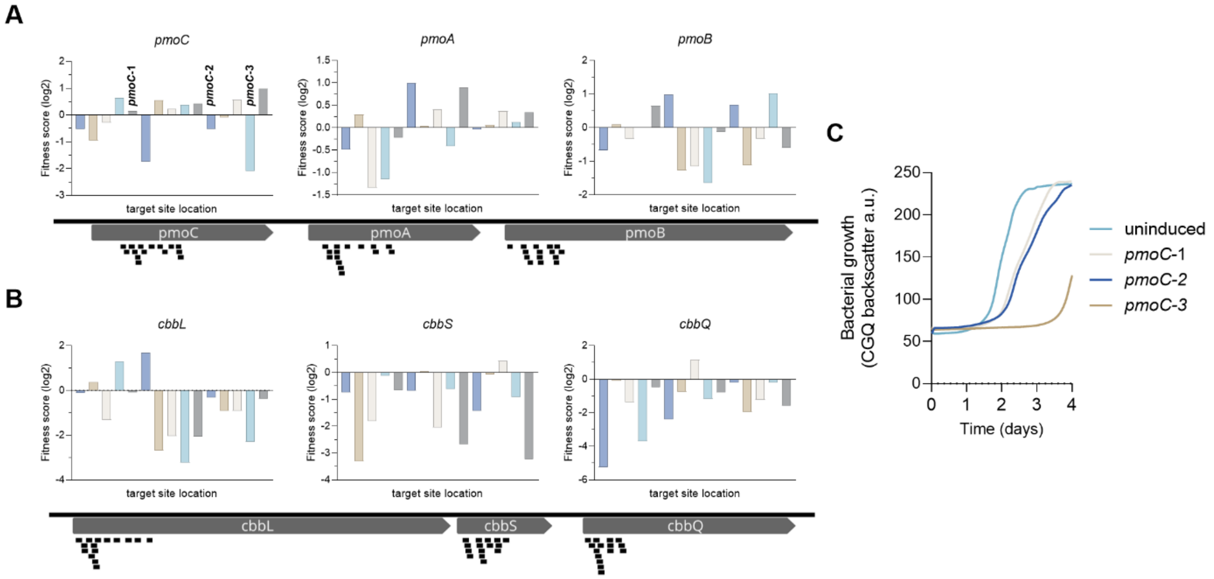

Figure S2. Some sgRNAs targeting the same gene have variable differential fitness scores. Mapped sgRNA target sequences (black rectangles) and corresponding differential fitness score within the *M. capsulatus* A) *pmoCAB* and B) *cbbLSQ* operon genes encoding the particulate methane monooxygenase and ribulose-1,5-bisphosphate carboxylase/oxygenase (RubisCO) and associated RubisCO activase, respectively. C) High resolution CH_4-dependent growth curves of strains with sgRNAs with differential fitness scores (*pmoC-1*, *pmoC-2*, *pmoC-3*) targeting the *pmoC* gene with (blue line) or without (purple line) CRISPRi induction. The growth curves represent the mean of 4-6 independent observations. The mean of the three uninduced sgRNA controls is shown.

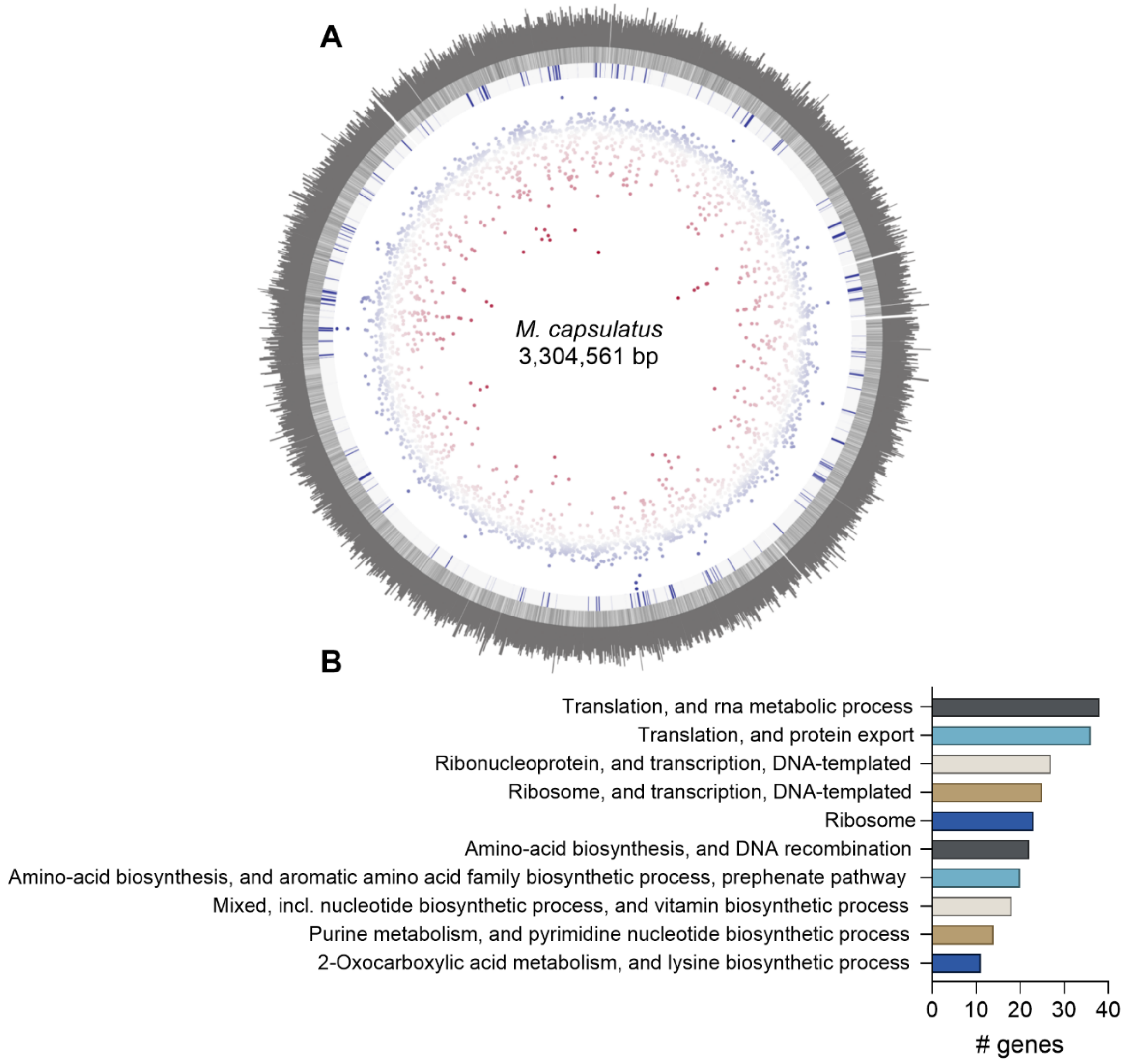

Figure S3. A CRISPRi genome-wide screen identifies genes essential for CH_4-dependent growth of *M. capsulatus*: mapping to the *M. capsulatus* genome. A) Circos plot of the methanotroph sgRNA library raw reads (outer ring) mapped to the NC_002977 *M. capsulatus* reference genome (2nd ring, genes in forward direction are in dark grey and reverse direction in light grey). Differential sgRNA fitness scores determined via DeSeq2 between CRISPRi induced and uninduced samples during selection/cultivation with CH_4 as the sole carbon and energy source (4th ring, blue dots log2 >1; red dots log2 <1). sgRNAs depleted in the screen (negative fitness scores) correspond to genes with transcription repression causing a growth defect where those enriched (positive fitness scores) correspond to genes with transcription repression causing a growth advantage. sgRNAs with statistically significant ($p \leq 0.01$) differential fitness scores are indicated (blue squares, 3rd ring). B) ShinyGo enrichment analysis of the 233 genes with sgRNA log2 fitness scores ≤ -1 and $p \leq 0.001$.

Data S1. (separate file)

Legend for table S1: sgRNA library sequences.

Legend for table S2: sgRNA library spacer sequences.

Legend for table S3: sgRNAs per gene counts.

Legend for table S4: Extracted sgRNA target sequence read counts mapped to the sgRNA library.

Legend for table S5: Extracted sgRNA target sequence read counts mapped to the *M. capsulatus* Bath genome.

Legend for table S6: Extracted sgRNA target sequences from CRISPRi uninduced replicate 1.

Legend for table S7: Extracted sgRNA target sequences from CRISPRi uninduced replicate 2.

Legend for table S8: Extracted sgRNA target sequences from CRISPRi uninduced replicate 3.

Legend for table S9: Extracted sgRNA target sequences from CRISPRi uninduced replicate 4.

Legend for table S10: Extracted sgRNA target sequences from CRISPRi induced replicate 1.

Legend for table S11: Extracted sgRNA target sequences from CRISPRi induced replicate 2.

Legend for table S12: Extracted sgRNA target sequences from CRISPRi induced replicate 3.

Legend for table S13: Extracted sgRNA target sequences from CRISPRi induced replicate 4.

Legend for table S14: Differential sgRNA fitness scores calcuated from reads mapped to the sgRNA reference library.

Legend for table S15: ShinyGO enrichment analysis of gene targets with differential sgRNA fitness identifed after mapping to the sgRNA reference library.

Legend for table S16: Differential sgRNA fitness scores calcuated from reads mapped to the *M. capsulatus* Bath genome.

Legend for table S17: ShinyGO enrichment analysis of gene targets with differential sgRNA fitness identifed after mapping to the *M. capsulatus* Bath genome.

Table S18. Strains and Plasmids

Name	Genotype	Source
***Methylococcus capsulatus* Bath**	Wild-type	Lab stock (originating from Kalyuzhnaya lab)
**CH2236**	*M. capsulatus* Bath with pDS1	(*48*)
**CH2484**	*M. capsulatus* Bath with pDS1 and pJHCi	This study
**CH2486**	*M. capsulatus* Bath with pJH1 and pJHCi^{sfgfp}	This study
**CH2342**	*M. capsulatus* Bath with inducible dCas9 integrated into the chromosome	This study
**CH2475**	CH2342 with p01sgRNA^{mrfp}	This study
**CH2473**	CH2342 with p01sgRNA^{cbbL}	This study
**CH2529**	CH2342 with p01sgRNA^{gwlibrary}	This study
**CH2537**	CH2342 with p01sgRNA^{MCA2127}	This study
**CH2477**	CH2342 with p01sgRNA^{MCA3033}	This study
**CH2535**	CH2342 with p01sgRNA^{MCA0037}	This study
**CH2476**	CH2342 with p01sgRNA^{MCA2470}	This study
**CH2536**	CH2342 with p01sgRNA^{MCA0567}	This study
**CH2598**	CH2342 with p01sgRNA^{MCA2302}	This study
**CH2474**	CH2342 with p01sgRNA^{pmoC-1}	This study
**CH2478**	CH2342 with p01sgRNA^{pmoC-2}	This study
**CH25104**	CH2342 with p01sgRNA^{pmoC-3}	This study
***Methylosinus trichosporium* OB3b**	Wild-type	(*18*)
**CH2507**	*M. trichosporium* OB3b with pDS1	(*48*)
**CH2508**	*M. trichosporium* OB3b with pDS1 and pJHCi	This study
***Escherichia coli* Zymo 10B**	F- *mcrA* Δ(*mrr-hsdRMS-mcrBC*) Φ80*lacZ*ΔM15 Δ*lacX*74 *recA1 endA1 araD139* Δ(*ara leu*) 7697 *galU galK rpsL nupG*	Zymo Research
***E. coli* S17-1**	Tp^r Sm^r *recA thi pro hsd*(r^-m^+)RP4-2-Tc::Mu::Km Tn7	ATCC 47055
**Plasmids**		
**Name**	**Description**	**Source**
**pCAH01^{SpR}**	IncP broad-host-range inducible expression plasmid	Addgene #128159(*44*)
**pCAH01^{SpR}::Cas9**	Inducible Cas9 expression	Addgene #128160(*44*)
**pdCas9-bacteria**	aTc-inducible expression of catalytically inactive *S. pyogenes* Cas9 (dCas9)	Addgene #44249(*40*)
**pDS1**	pBBR1MCS-5 with BBa_J23119-mRFP reporter	(*48*)

**pJHCi**	pCAH01^{SpR}::Cas9 *cas9* gene replaced with *dcas9* and *M. capsulatus* P_{mxa} driving expression of mRFP targeting sgRNA	This study
**pJHint**	*tetO*-TetR inducible suicide plasmid that integrates into the *M. capsulatus* genome between MCA and MCA.	This study
**pJHint::dCas9**	pJHint with dCas9 gene for chromosomal integration of inducible dCas9 cassette	This study
**pLSQ**	*M. capsulatus cbbLSQ* operon with native promoter cloned into pBMTL-2	This study
**pL$^{PAMC>T}$SQ**	pCbbLSQ with mutated PAM site	This study
**p01sgRNA^{mrfp}**	pCAH01 with sgRNA targeting mRFP ORF	This study
**p01sgRNA^{cbbL}**	pCAH01 with sgRNA targeting MCA2743 (*cbbL*) ORF	This study
**p01sgRNA^{MCA2127}**	pCAH01 with sgRNA targeting MCA2127 (*asnB*) ORF	This study
**p01sgRNA^{MCA3033}**	pCAH01 with sgRNA targeting MCA3033 (*dnaA*) ORF	This study
**p01sgRNA^{MCA0037}**	pCAH01 with sgRNA targeting MCA0037 (*edd*) ORF	This study
**p01sgRNA^{MCA2470}**	pCAH01 with sgRNA targeting MCA2470 (*fdx*) ORF	This study
**p01sgRNA^{MCA0567}**	pCAH01 with sgRNA targeting MCA0567 (*rpsB*) ORF	This study
**p01sgRNA^{MCA2302}**	pCAH01 with sgRNA targeting MCA2301 (*cbtB*) ORF	This study
**p01sgRNA$^{MCA1798/2855}$**	pCAH01 with sgRNA targeting MCA (*pmoC-1*) ORF	This study
**p01sgRNA$^{MCA1798/2855}$**	pCAH01 with sgRNA targeting MCA (*pmoC-2*) ORF	This study
**p01sgRNA$^{MCA1798/2855}$**	pCAH01 with sgRNA targeting MCA (*pmoC-3*) ORF	This study

Table S19. Primers and synthetic DNA fragments

Name	Sequence
	Construction of pJHCi
**oCAH230 pCAH01^{SpR}::Cas9 F**	GTTCTTTCCTGCGTTATCCC
**oCAH231 pCAH01^{SpR}::Cas9 R**	GTCTGACGCTCAGTGGAA
**oCAH267 P$_{mxa}$-sgRNA F**	CGTTCCACTGAGCGTCAGACGAGGTTCAGGCGAAACCG
**oCAH268 P$_{mxa}$-sgRNA R**	GGGATAACGCAGGAAAGAACAAAAAAAGCACCGACTCGGT
**oCAH4 pCAH01 F**	AAGCTTGACCTGTGAAGTG
**oCAH3 pCAH01 R**	TTCACTTTTCTCTATCACTGATAG
**oCAH952 dCas9 F**	gtgatagagaaaagtgaaATGGATAAGAAATACTCAATAGGCTTAGCT
**oCAH1507 dCas9 R**	cttcacaggtcaagcttTCAGTCACCTCCTAGCTGACTCAAAT
	Construction of pJHint and M. capsulatus strain with chromosomal dCas9 integration
**oCAH403 part 1 F**	gcagcggaaaagCCCGTAGAAAAGATCAAAGG
**oCAH404 part 1 R**	cggatcgtgcgcATGTGAGCAAAAGGCCAG
**oCAH405 part 2 F**	cttttgctcacatGCGCACGATCCGTATTCC
**oCAH406 part 2 R**	ttaggaattaatcATCTGTCGAAGCGTGCCG
**oCAH407 part 3 F**	gcttcgacagatGATTAATTCCTAATTTTTGTTGAC
**oCAH408 part 3 R**	aacgagacatcatACTCTTCCTTTTTCAATATTATTG
**oCAH409 part 4 F**	aaaaggaagagtATGATGTCTCGTTTAGATAAAAGTAAAGTGATTAACAG
**oCAH410 part 4 R**	caagggccttgcGCGCGCTCTACGAACTGC
**oCAH411 part 5 F**	tcgtagagcgcgcGCAAGGCCCTTGGCTCTTG
**oCAH412 part 5 R**	cttcgcggagctATCTCTCGCAAGACGGCG
**oCAH413 part 6 F**	tcttgcgagagatAGCTCCGCGAAGTCGCTC
**oCAH414 part 6 F**	tcttttctacgggCTTTTCCGCTGCATAACCCTG
**oCAH447 F**	GACGACGACCGTCACAAA
**oCAH448 R**	GTTTTCGTGATTCGCCGC
	Construction of pLSQ and MCA2743(cbbL) PAM mutation
**oCAH1007 pDS1 F**	GGATCCAAACTCGAGTAAG
**oCAH16 pDS1 R**	TAGCCGCTTATGTCTATTGCTG
**oCAH1629 P$_{cbbL}$ F**	cagcaatagacataagcggctaTTATGCTAATCGCCCTTCATCCG
**oCAH1750 *cbbQ* R**	gagatccttactcgagtttggatccTCAGAAAAACGTCGCTACGG
**oCAH1751 pLSQ F**	TGACCGACCTCGACTATTAC
***cbbL*:c.57C>T synthetic fragment**	CGGTAAACCAGCAATAGACATAAGCGGCTATTATGCTAATCGCCCTTCATCCGGCGGTAGCAGGCAGGCATATCAGTATAATGCGCGCTTATTGTCGTGATTATGACAATTTAGTTCCGTTTATGGATATCAGCGCTTATAGTGCCTCGCGAAAAGGGATCGCCCCGATGCCGGCTAGGGAACGGCGGTTCATTGCCATAGCGCTGTTGCAAAACGAGTAGGAGAAAACCATGGCTGTCAAAACATACAACGCGGGCGTCAAGGAATACCGCGAAACCTACTGGGATCCGAACTACACTCCCGCCGACACCGATCTGCTGGCGGTCTTCAAGATCACCCCTCAGCCGGGTGTG

	CCGCGCGAAGAAGCCGCCGCCGCCGTGGCCGCGGAATCG TCGACCGGCACCTGGACCACCGTCTGGACCGACCTGCTG ACCGACCTCGACTATTACAAGGGCCGCG
sgRNA exchange and sgRNA library generation	
**oCAH1083 F**	taccactccctatcagtgatTGGAACCGTACTGGAACTGC
**oCAH1081 R**	cttcacaggtcaagcttAAAAAAAGCACCGACTCGGT
**oCAH1190 01*sgRNA*^{mrfp} F**	GTTTTAGAGCTAGAAATAGCAAGTTAAAATAAGGCT
**oCAH119 01*sgRNA* R**	ATCACTGATAGGGAGTGGTAAAATAACTCTATCAATGAT AGAG
**oCAH1084 *sgRNA*^{cbbL} oligo**	TACCACTCCCTATCAGTGATCATTGTCGGCGGGAGTGTAG TTCGTTTTAGAGCTAGAAATAGCAAGTT
**oCAH1735 *sgRNA*^{MCA0037} oligo**	TACCACTCCCTATCAGTGATTGAGATCGAGGTAAGCGGC GGTTTTAGAGCTAGAAATAGC
**oCAH1736 *sgRNA*^{MCA0567} oligo**	TACCACTCCCTATCAGTGATCATCTGACGCATGGTAACTG GTTTTAGAGCTAGAAATAGC
**oCAH1737 *sgRNA*^{MCA2127} oligo**	TACCACTCCCTATCAGTGATGGGCCAGGCGCTCGATCATG GTTTTAGAGCTAGAAATAGC
**oCAH1738 *sgRNA*^{MCA2470} oligo**	TACCACTCCCTATCAGTGATGCAGTTTTCGGTCACAACAA GTTTTAGAGCTAGAAATAGC
**oCAH1739 *sgRNA*^{MCA3033} oligo**	TACCACTCCCTATCAGTGATCAGGTGTTGAACTGTTGCGG GTTTTAGAGCTAGAAATAGC
**oCAH1893 *sgRNA*^{MCA2302} oligo**	TACCACTCCCTATCAGTGATCGACATGGACCGGCAAGGA AGTTTTAGAGCTAGAAATAGC
**oCAH1082 *sgRNA*^{pmoC1} oligo**	TACCACTCCCTATCAGTGATCGCCGCAGCTGCACCACCGT TTTAGAGCTAGAAATAGC
**oCAH1740 *sgRNA*^{pmoC2} oligo**	TACCACTCCCTATCAGTGATCCGCACCCACAGGTAGAAC AGTTTTAGAGCTAGAAATAGC
**oCAH1905 *sgRNA*^{pmoC3} oligo**	TACCACTCCCTATCAGTGATGTACAGGAAATTCATCCAGT GTTTTAGAGCTAGAAATAGC
**oCAH1416 sgRNA^{library} F**	GTTGACACTCTATCATTGATAGA
**oCAH1417 sgRNA^{library} R**	TGATAACGGACTAGCCTTATTTT

Lowercase sequence are homology arms for isothermal assembly. Bold sequence is the sgRNA target sequence
